# PAK1 Regulates MEC-17 Acetyltransferase Activity and Microtubule Acetylation during Proplatelet Extension

**DOI:** 10.3390/ijms21207531

**Published:** 2020-10-13

**Authors:** Juliette van Dijk, Guillaume Bompard, Gabriel Rabeharivelo, Julien Cau, Claude Delsert, Nathalie Morin

**Affiliations:** 1 Université de Montpellier, 34293 Montpellier, France; guillaume.bompard@crbm.cnrs.fr (G.B.); gabriel.rabeharivelo@crbm.cnrs.fr (G.R.); julien.cau@igh.cnrs.fr (J.C.); claude.delsert@crbm.cnrs.fr (C.D.); 2 CRBM, CNRS, UMR 5237, 1919 Route de Mende, 34293 Montpellier, France; 3 IGH, CNRS UMR 9002, 141, Rue de la Cardonille, 34396 Montpellier, France; 4 Montpellier Rio Imaging, 34293 Montpellier, France; 5 3AS Station Expérimentale d’Aquaculture Ifremer, Chemin de Maguelone, 34250 Palavas-les-Flots, France

**Keywords:** microtubules, acetylation, megakaryocytes, proplatelet, p21-activated kinase 1 PAK1, acetyltransferase MEC-17

## Abstract

Mature megakaryocytes extend long processes called proplatelets from which platelets are released in the blood stream. The Rho GTPases Cdc42 and Rac as well as their downstream target, p21-activated kinase 2 (PAK2), have been demonstrated to be important for platelet formation. Here we address the role, during platelet formation, of PAK1, another target of the Rho GTPases. PAK1 decorates the bundled microtubules (MTs) of megakaryocyte proplatelets. Using a validated cell model which recapitulates proplatelet formation, elongation and platelet release, we show that lack of PAK1 activity increases the number of proplatelets but restrains their elongation. Moreover, in the absence of PAK1 activity, cells have hyperacetylated MTs and lose their MT network integrity. Using inhibitors of the tubulin deacetylase HDAC6, we demonstrate that abnormally high levels of MT acetylation are not sufficient to increase the number of proplatelets but cause loss of MT integrity. Taken together with our previous demonstration that MT acetylation is required for proplatelet formation, our data reveal that MT acetylation levels need to be tightly regulated during proplatelet formation. We identify PAK1 as a direct regulator of the MT acetylation levels during this process as we found that PAK1 phosphorylates the MT acetyltransferase MEC-17 and inhibits its activity.

## 1. Introduction

Platelets are small anucleate cytoplasts derived from giant precursor cells, the megakaryocytes (MKs), residing in the bone marrow. MK maturation is initiated by multiple rounds of endomitosis, the synthesis of platelet-specific granules, and the formation of a demarcation membrane system (DMS) required for forming the plasma membranes of the future platelets [1]. Mature MKs extend long protrusions called proplatelets (PPL) into the sinusoidal vessel lumen, and the tips of these protrusions are shed by shear forces and release preplatelets or platelets in the blood stream [2,3]. The F-actin and microtubule (MT) cytoskeletons are both required for PPL formation. While F-actin is involved in the branching of PPL shafts, thereby increasing the number of PPL tips available for platelet release [4], MTs provide the driving force for PPL formation. They form bundles which slide up against each other, in a dynein-dependent manner, providing the pushing force to generate initial broad pseudopodia and elongate them to form PPLs [5,6]. At the PPL tips, MT bundles turn around forming loops that will give rise to the MT ring known as the marginal band which forms the skeleton of the platelets. This MT ring is mainly composed of β1-tubulin, a hematopoietic-specific beta-tubulin isoform expressed only in mature megakaryocytes. β1-tubulin is mandatory for PPL formation [7] and accordingly, TUBB1 mutations in human were found associated with macrocytopenia [8,9,10,11]. We recently showed that two MT post-translational modifications, acetylation and polyglutamylation, occur in PPLs and are also mandatory for PPL formation [12].

The various members of the Rho family of small GTPases are critical upstream regulators of both actin and MT cytoskeletons [13]. The specific contribution of Rac and Cdc42 during MK maturation and platelet production was studied in mice lacking these proteins in the MK lineage. Whereas deletion of either Rac or Cdc42 had no or only mild effect on platelet counts, combined depletion of both Rho GTPases resulted in severe macrothrombocytopenia [14]. The MKs in double-deficient mice exhibited a highly abnormal morphology with normal ploidy but few demarcation membranes and an overall reduction in granule numbers. Moreover, Rac and Cdc42 deficiency abrogated PPL formation. Strikingly, this phenotype was associated with severely defective tubulin organization, whereas actin assembly and structure were barely affected. These results suggested that the combined action of Rac and Cdc42 is crucial for platelet production, particularly by regulating microtubule dynamics.

The p21-activated kinases (PAKs) are serine/threonine kinases known to act downstream of Rac and Cdc42. They regulate both actin and MT networks through phosphorylation of a large number of substrates (for review see [15]). MKs express all members of the group I PAKs (PAK1, PAK2 and PAK3). Bone marrow depletion of PAK2 is associated with macrocytopenia [16]. PAK2 was shown to act in multiple steps of megakaryopoiesis as its lack increases MK polyploidization and alters DMS as well as PPL formation [16]. Depletion of PAK1 or PAK3 did not induce obvious defects in blood composition [16,17] and therefore, their functions in platelet formation have not been studied. However, group I PAKs might have redundant functions as evidenced earlier in the brain. Indeed, despite their well-documented neuronal functions (reviewed in [18,19]), single knockout mice for PAK1 and PAK3 exhibit no abnormalities in overall neuronal morphology and brain development [17,20] while PAK1 and PAK3 double knockout mice showed major loss of brain volume [21].

Here, we address the role of PAK1 specifically in platelet formation using a recently characterized cellular model which mimics PPL formation and platelet release [12]. This CHO-based cell line constitutively expresses a partially active mutant α2bβ3 integrin (D723H cells). Upon fibrinogen binding, these cells express the hematopoietic-specific β1-tubulin and extend PPL-like extensions (PPLLs). In the present study, we found that PAK1 regulates proplatelet formation. In its absence, cells display increased MT acetylation levels, loose MT integrity and fail to form correctly elongated proplatelets. We show that PAK1 directly phosphorylates the tubulin acetyltransferase MEC-17 and inhibits its activity. Thus, PAK1 is required to maintain a correct MT acetylation level during PPL formation.

## 2. Results

### 2.1. PAK1 Colocalizes with Microtubules and Actin in Proplatelet Extensions

**Figure 1:**
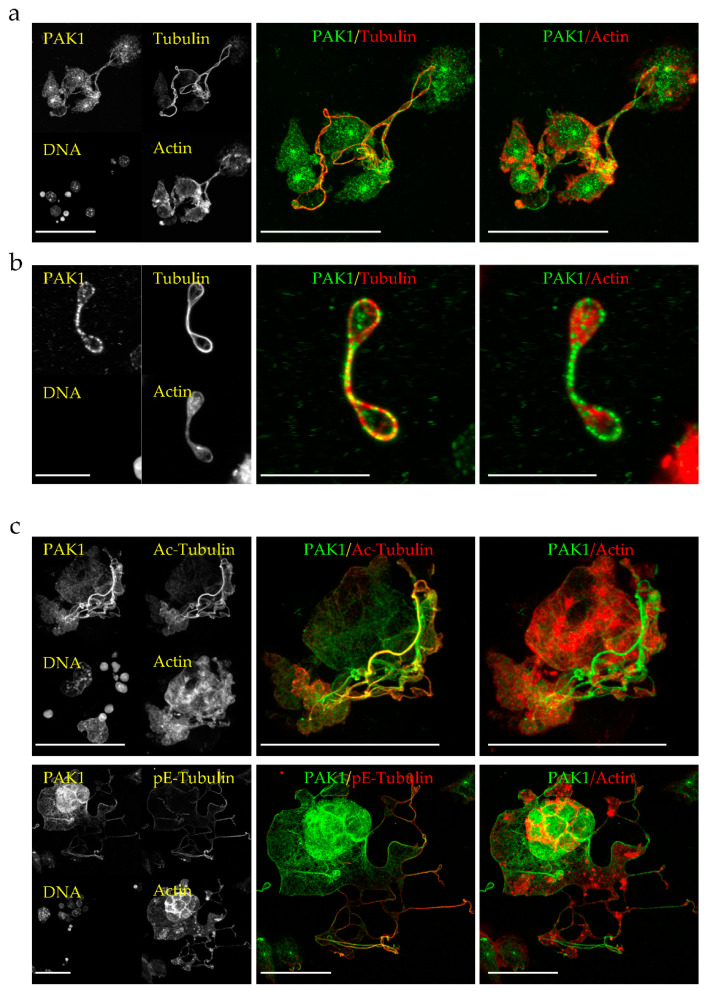
Endogenous PAK1 decorates microtubules (MTs) and actin in megakaryocyte proplatelets (PPL) extensions and in preplatelets. (**a**,**b**) Representative MIP images of E13.5 mouse embryo liver cells-derived megakaryocytes extending PPLs (**a**) and released preplatelets (**b**) stained for endogenous PAK1, total tubulin and F-actin. Bars are 50 µm (**a**) and 10 µm (**b**). (**c**) Representative MIP images of E13.5 mouse embryo liver cells-derived megakaryocytes extending PPL-like extensions (PPLLs) stained with PAK1, F-actin and either acetylated-tubulin (Ac-Tubulin) or polyglutamylated-tubulin (pE-Tubulin). Bars are 50 µm. All images were acquired on a Leica SP8-UV confocal microscope with a 63x HCX Plan APO 1.4NA oil CS2 objective.

**Figure 2:**
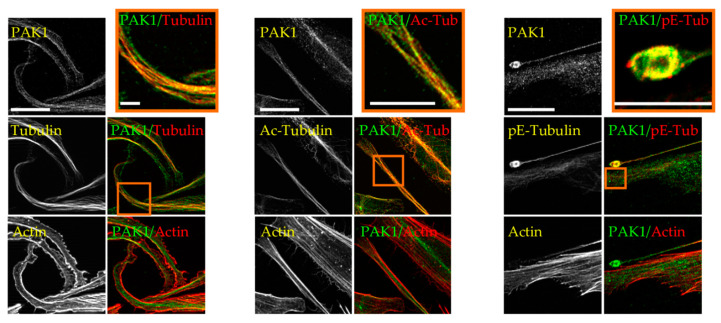
Endogenous PAK1 decorates MTs and actin in PPLL extensions of D723H cells. Representative MIP images of PPLL extensions of D723H cells spread on fibrinogen for 16 h and stained for endogenous PAK1, F-actin and either total tubulin, acetylated-tubulin (Ac-Tub) or polyglutamylated-tubulin (pE-Tub). Bars are 20 µm. Orange frames (high magnification insets) are single confocal slices (Bars are 10µm). All images were acquired on a Leica SP8-UV confocal microscope with a 63× HCX Plan APO 1.4NA oil CS2 objective.

To determine the cellular localization of PAK1 in MKs, we performed immunofluorescence of endogenous PAK1 in mouse embryonic liver-derived megakaryocytes. In PPL extensions of mature megakaryocytes as well as in the released barbell-shaped preplatelets, endogenous PAK1 colocalizes with both the actin and microtubule networks (Figure 1a,b). These results were confirmed by analysis of the colocalized voxels between PAK1 and microtubules or PAK1 and actin on selected regions of the3D images using Imaris software and by the calculated Pearson’s correlation coefficients (Figure S1a,b). Since PPL MTs undergo acetylation and polyglutamylation [12], we wondered whether PAK1 binds to a specifically modified MT subtype but found that PAK1 decorates both acetylated and polyglutamylated MTs (Figure 1c and Figure S1c). PAK1 has a similar localization in D723H cells plated on fibrinogen as it colocalizes with MTs and actin in PPLL extensions, is present in the MT ring formed at the tips of the extensions, and decorates both acetylated and polyglutamylated MTs (Figure 2).

### 2.2. Loss of PAK1 Kinase Activity Induces the Formation of Many Short PPLLs, Increases MT Acetylation and Impairs MT Integrity

**Figure 3:**
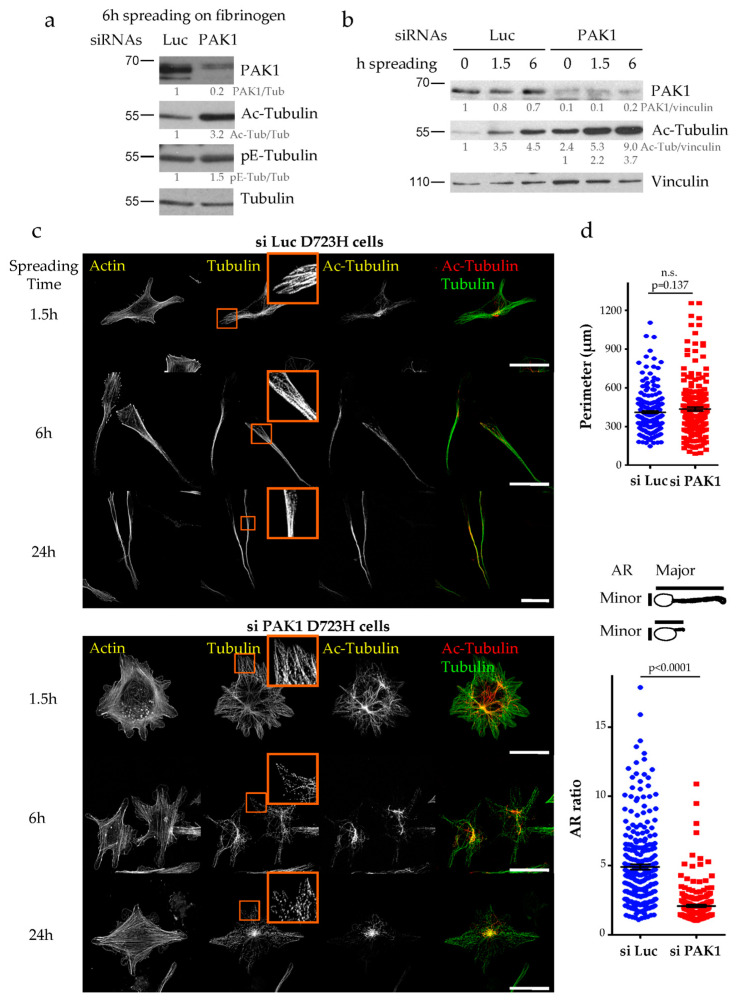
Loss of PAK1 induces the formation of multiple short protrusions, increases MT acetylation and disrupts MT integrity. (**a**,**b**) Luc and PAK1-depleted D723H cells were starved for 2 h, 48 h post transfection and spread on fibrinogen for the given number of hours. Total cell extracts were analyzed by western blot. Protein bands were quantified by densitometry and normalized using tubulin (**a**) or vinculin (**b**). Values below protein bands represent their relative abundance. (**c**). MIP of representative images of Luc and PAK1-depleted D723H cells stained for actin, tubulin and acetylated-tubulin (Ac-Tub) at different times of spreading on fibrinogen. Bars are 30 µm. Orange frames are high magnifications of 15 × 15 µm². (**d**). Quantification of the cell perimeter and AR ratio (ratio of the cell major/minor axis) were performed after 6 h of spreading on fibrinogen. A total of 259 si Luc and 208 si PAK1 cells were counted from three independent experiments. Statistical calculations were performed using a two-tailed unpaired Student’s *t* test. *p* *<* 0.0005 is considered significant. n.s. stands for non-significant. Error bars are SEM.

To address the role of PAK1 during PPL extension, we used an siRNA approach in D723H cells. PAK1-depleted or control Luciferase-depleted cells were spread on fibrinogen to induce PPLL formation and analyzed at different time points. As shown by western blot (Figure 3a) and immunofluorescence (Figure S2), PAK1 is efficiently depleted (80–90% depletion). After 6 h spreading, PAK1-depleted cells have significantly higher amount of acetylated tubulin but not polyglutamylated tubulin as compared to control Luciferase-depleted cells (Figure 3a and Figure S2). We previously showed that MT acetylation increases during the time course of D723H cells spreading on fibrinogen [12]. In PAK1-depleted cells, the MT acetylation level still increases but the initial level is two to three times higher (Figure 3b). Actin staining at different times after spreading on fibrinogen showed that while control cells formed one or two long PPLLs, PAK1-depleted cells form multiple protrusions (Figure 3c). While the total cell perimeter of PAK1-depleted cells is almost not affected, measurement of the ratio of the cell major/minor axis (AR ratio) shows that the protrusions formed in the absence of PAK1 do not elongate as well as in control cells (Figure 3d). In addition to the formation of multiple PPLLs that poorly elongate, live cell imaging shows that some PAK-1 depleted cells that started to elongate PPLLs die after retracting their PPLLs (Supplementary Movies 1–2). One possible cause of cell death is revealed by observation of the MT network (Figure 3c). After 1.5 h spreading, PAK1-depleted cells have nice MTs that are almost all acetylated. However, at later time points the MT network appears as small fragments as if they have been broken or severed (Figure 3c, insets). Taken together, we found that PAK1-depleted cells produce multiple protrusions that do not elongate and that their MT network is hyperacetylated and loses its integrity during PPLL formation.

**Figure 4:**
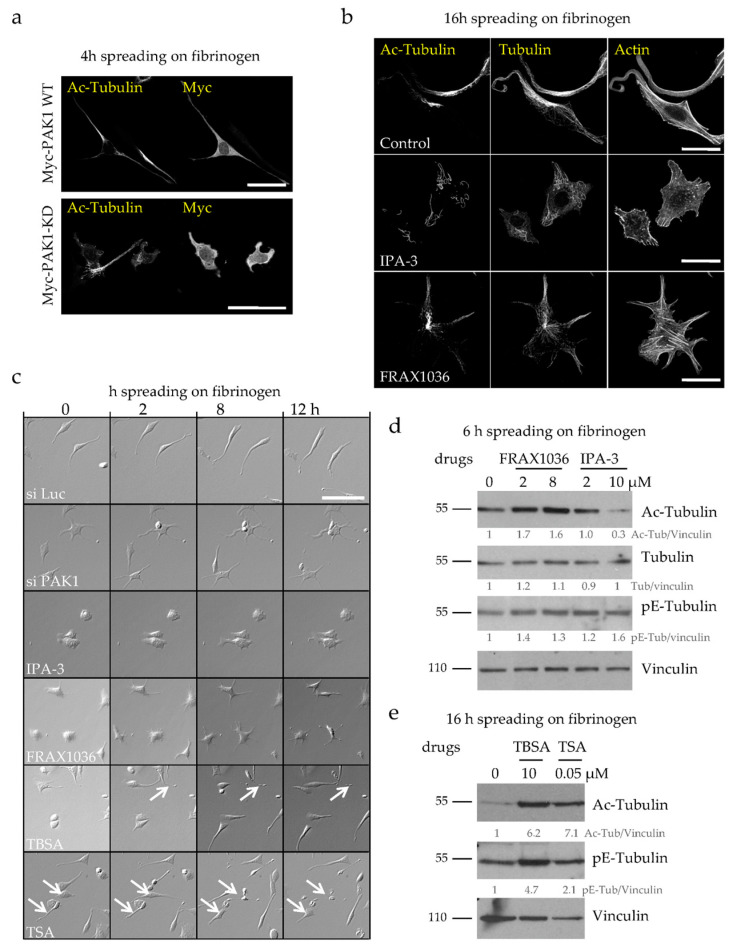
The kinase activity of PAK1 is required for its action on MT acetylation, MT integrity and PPLL formation. (**a**) Representative MIP images of Myc-PAK1 and Myc-PAK1-KD transfected D723H cells (24 h) followed by serum starvation and spreading for 4 h on fibrinogen. Bars are 50 µm. (**b**) Representative MIP images of D723H cells treated 30 min after spreading on fibrinogen with vehicle (control) or IPA-3 (5 µM) or FRAX1036 (2 µM) for 16 h and stained for acetylated-tubulin (Ac-Tub), total tubulin and actin. Bars are 30 µm. (**c**) Phase contrast and wide field images of Luc-depleted, PAK1-depleted or drug-treated D723H cells were acquired at indicated times, bars are 100 µm (see also Supplementary Movies 1–6). (**d**,**e**). Western blot analyses of D723H cell extracts treated with the given drug concentrations and analyzed with indicated antibodies after 6 h (**d**) or 16 h (**e**) spreading on fibrinogen. Protein bands were quantified by densitometry and normalized to tubulin. Values below protein bands represent their relative abundance.

To determine the contribution of the kinase activity of PAK1 to the phenotypes observed after PAK1 depletion, we expressed a dominant negative kinase dead PAK1 mutant (PAK1-KD which has a K/R substitution in the ATP binding site) in D723H cells. While transient expression of wild type PAK1 did not affect PPLL elongation, PAK1-KD expression induced the formation of several short protrusions similarly to PAK1 depletion (Figure 4a). Thus, loss of the kinase activity of PAK1 is sufficient to mimic the phenotype. To confirm this result, we used the PAK inhibitors IPA-3 and FRAX1036. D723H cells treated with IPA-3, which allosterically prevents Cdc42 binding to PAK1 [22], formed several protrusions but live imaging showed that these protrusions resembled lamellipodia rather than short PPLLs (Figure 4b,c; Supplementary Movie 3). We also observed massive MT breakage and depolymerization (Figure 4b), a phenotype not yet reported for this inhibitor. Immunoblots reveal that although the remaining MTs are entirely acetylated (Figure 4b), the acetyl-tubulin reactivity is reduced (Figure 4d). This likely reflects an increase in the amount of tubulin heterodimers which are preferential substrates for HDAC6 deacetylase and are thus rapidly deacetylated. We cannot rule out either that IPA-3 may target other substrates responsible for massive MT depolymerization and deacetylation. FRAX1036 is an ATP competitive inhibitor and displays good selectivity towards both PAK1 and PAK2 [23]. FRAX1036-treated D723H cells accurately mimicked PAK1 depletion, inducing several short protrusions that resemble those observed in PAK1-depleted cells (Figure 4b,c; Supplementary Movie 4). Moreover, like PAK1 depletion, FRAX1036 induces an increase in MT acetylation (Figure 4d). Thus, both PAK1-KD expression and FRAX1036 treatment faithfully reproduce PAK1 depletion demonstrating that the kinase activity of PAK1 is sufficient for its action on the number of formed protrusions, PPLL elongation, MT acetylation and MT integrity.

### 2.3. MT Hyperacetylation Does Not Increase the Number of PPLLs but Impairs MT Integrity

We next determined whether the increase in MT acetylation in PAK1-depleted cells is the cause of the increased number of protrusions and/or loss of MT integrity and cell death. For this, we treated D723H cells with inhibitors of HDAC6, the main MT deacetylase. Both tubastatin- and TSA-treatment increase the acetyl-tubulin level (Figure 4e). Live cell imaging showed that during the initial spreading, most drug-treated cells behaved like controls, forming only one or two long PPLL extensions (Figure 4c). However, after 8 to 10 h spreading, many PPLL extensions retracted and cell death occurred (Figure 4c, Supplementary Movies 5–6). Thus MT hyperacetylation is not sufficient to cause the increase in the number of protrusions, but contributes to loss of MT integrity and cell death.

### 2.4. PAK1 Does Not Affect the Activity of the Tubulin Deacetylase HDAC6

**Figure 5:**
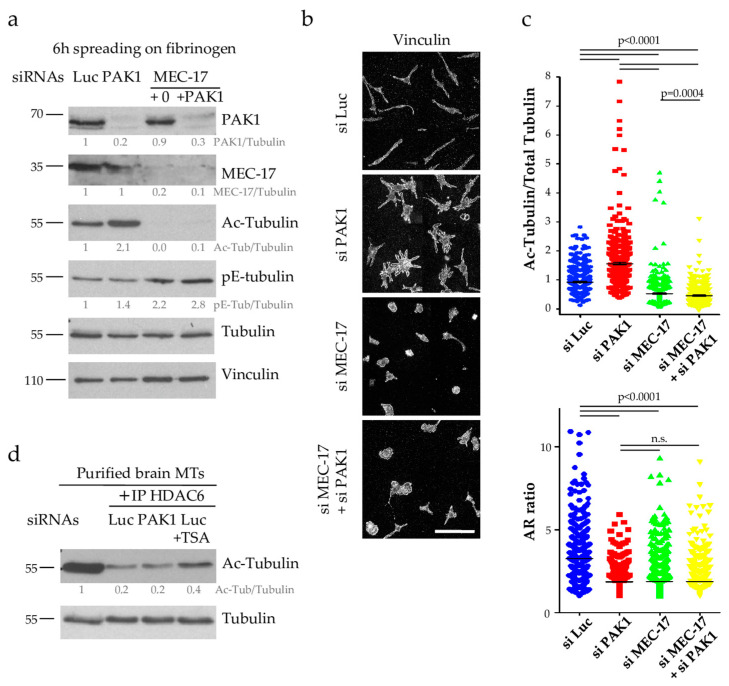
PAK1 does not regulate the HDAC6 pathway (**a**) Total protein extracts of Luc, PAK1, MEC-17 and MEC-17+PAK1 depleted D723H cells (using partial MEC-17 depletion, 15 nM siRNA) were analyzed by western blots with indicated antibodies. Protein bands were quantified by densitometry and normalized to tubulin. Values below protein bands represent their relative abundance. (**b**) Immunofluorescence images at low magnification (mount image of wide field ×40, scale bar is 100 µm) of the cells treated as in (**a**). (**c**) Quantification of Acetyl-tubulin/Total tubulin ratio and AR ratio of cells treated as in (**a**) and immunostained with anti-tubulin, anti-acetylated tubulin or vinculin antibodies. The number of cells analyzed varies from 441 to 845 and were obtained from three independent experiments. Statistical calculations were performed using a two-tailed unpaired Student’s *t* test. *p* *<* 0.0005 is considered significant. n.s. stands for non-significant. Error bars are SEM. (**d**) Total protein extracts of Luc or PAK1-depleted and Luc-depleted/TSA-treated D723H cells were immunoprecipitated with HDAC6 antibodies and immunoprecipitates were incubated with purified brain MTs for 1 h and analyzed by western blot to determine the level of acetylated tubulin. Protein bands were quantified by densitometry and normalized to tubulin. Values below protein bands represent their relative abundance.

The fact that MT acetylation increases upon PAK1 depletion suggests that PAK1 has a role in downregulating MT acetylation. This can be achieved either by inhibiting the MT acetyltransferase MEC-17 or by activating the deacetylase HDAC6. Many regulators of HDAC6 activity have been described [24,25,26,27] and we thus first determined whether PAK1 affects the HDAC6 pathway. We have previously shown that the acetyltransferase MEC-17 is required for PPLL formation and that TSA-mediated inhibition of HDAC6 could rescue MT acetylation and PPLL formation in partial MEC-17-depleted D723H cells probably by stabilizing the small pool of acetylated tubulin [12]. We hypothesized that if PAK1 activates HDAC6 activity, its depletion should lower HDAC6 activity and thereby restore MT acetylation and eventually PPLL formation in partial MEC-17-depleted D723H cells. To test this, we depleted PAK1 together with partial MEC-17 depletion. PAK1 codepletion did not rescue the level of acetylated-tubulin nor the formation of PPLLs (Figure 5a–c) strongly suggesting that PAK1 does not act on the HDAC6 pathway. To confirm this result, we immunoprecipitated HDAC6 from D723H cells depleted of Luciferase or PAK1 and tested its deacetylase activity on pure brain MTs which are heavily acetylated. As a positive control, we also immunoprecipitated HDAC6 from control Luciferase-depleted cells treated with the HDAC6 inhibitor TSA. HDAC6 activity was high in immunoprecipitates from Luciferase-depleted cells as most brain MTs were deacetylated. TSA treatment reduced HDAC6 activity while PAK1-depleted cells behaved like Luciferase-depleted cells confirming that HDAC6 activity is not targeted by PAK1 (Figure 5d).

### 2.5. PAK1 Directly Phosphorylates MEC-17 and Inhibits Its Acetyltransferase Activity

**Figure 6:**
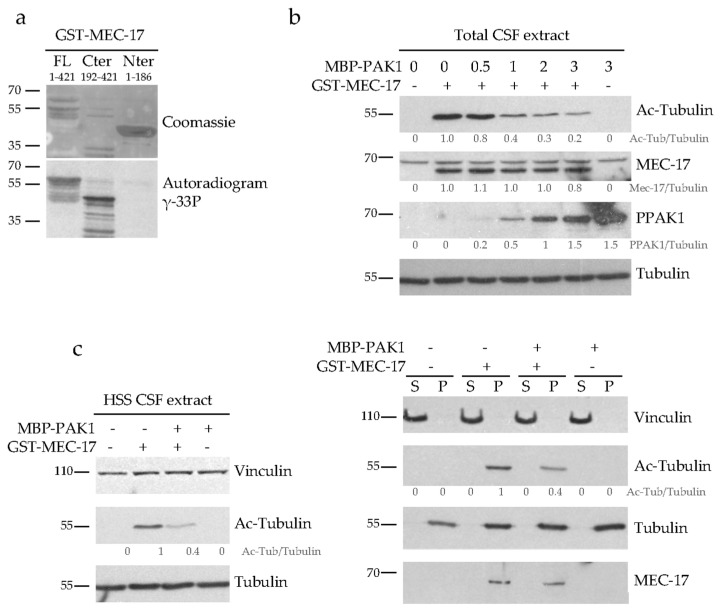
PAK1 phosphorylates MEC-17 and inhibits its acetyltransferase activity but not its binding to MTs. (**a**) In vitro phosphorylation assays of recombinant GST-MEC-17 (full length FL, N- or C-terminus) by recombinant MBP-PAK1 after incubation in the presence of γ-33P ATP were visualized by Coomassie staining and analyzed by autoradiography to detect γ-33P incorporation. (**b**) Purified recombinant GST-MEC-17 was phosphorylated in vitro by increasing concentrations of MBP-PAK1 in the presence of γ-S ATP (to avoid phosphatase-mediated dephosphorylation) and then added to a mitoticarrested Xenopus egg extract (CSF). Acetyltransferase activity was assessed by analyzing the acetylation level of the tubulin present in the CSF extract by western blot with the anti-acetyl-tubulin antibody. The fraction of active PAK1 was assessed using an anti-phospho PAK1 antibody (PPAK1). Note that non-phosphorylated GST-MEC-17 is active in CSF extract (first two lanes) while MBP-PAK1 by itself is not (last lane). (**c**) Western blots of HSS CSF extracts were analyzed with indicated antibodies following incubation with either recombinant GST-MEC-17, PAK1-phosphorylated-MEC-17 (+MBP-PAK1+GST-MEC-17) or PAK1 alone (left panel). The extracts were then centrifuged to pellet the MTs and both the soluble tubulin (S) and the MT pellet (P) fractions were analyzed by western blot with the indicated antibodies (right panel). All western blots were quantified by densitometry and normalized to tubulin. Values below protein bands represent their relative abundance.

Since PAK1 does not modulate the MT acetylation levels by regulating the HDAC6 pathway, we investigated its possible effect on MEC-17 activity. While many regulators of HDAC6 have been described, little is known about the mechanisms that regulate the activity of MEC-17. Since inhibition of the kinase activity of PAK1 is sufficient to increase MT acetylation, we tested whether PAK1 can directly phosphorylate MEC-17. Using an in vitro kinase assays with purified recombinant proteins, we found that PAK1 efficiently phosphorylated MEC-17 (Figure 6a). The C-terminal regulatory domain of MEC-17 (aa 192-421) is highly sensitive to proteases during the purification process but it is clearly phosphorylated by PAK1. In contrast the N-terminal catalytic domain of MEC-17 (aa 1-186) is not (Figure 6a). To identify putative PAK1 phosphorylation sites in the C-terminus of MEC-17, serine/threonine residues that match the PAK1 phosphorylation consensus sequence (T260/S270/S271/S272/S276/S315) were individually mutated into alanine and mutants were tested for their phosphorylation by PAK1. All mutants were still phosphorylated by PAK1, indicating that, at least in vitro, PAK1 targets several sites in MEC-17 (data not shown).

We next examined how PAK1-mediated phosphorylation of MEC-17 affects its acetyltransferase activity. MEC-17 was phosphorylated in vitro with increasing PAK1 concentrations and its activity was then tested in mitotic Xenopus egg extracts (CSF). These extracts contain tubulin that is not acetylated, at least not at a detectable level, but which can be efficiently acetylated by the addition of exogenous MEC-17 (Figure 6b, second versus first lane). The more PAK1 was used to phosphorylate MEC-17 prior to its addition to the extract, the less acetyl-tubulin it generated (Figure 6b, middle lanes). As a control we show that the highest concentration of PAK1 kinase by itself does not affect the acetyl-tubulin level when added to the extract (Figure 6b, last lane). This demonstrates that PAK1-mediated phosphorylation of MEC-17 downregulates its acetyltransferase activity.

To decipher the molecular mechanism by which PAK1-mediated phosphorylation inhibits the acetyltransferase activity of MEC-17, we tested the affinity of phosphorylated MEC-17 for its MT substrate. For this, high speed CSF extracts (HSS) were incubated with recombinant MEC-17, PAK1-phosphorylated-MEC-17 or PAK1. As previously, non-phosphorylated MEC-17 was more efficiently acetylating MTs than phosphorylated MEC-17 (Figure 6c). After inducing MT polymerization by adding taxol, we sedimented the MTs by centrifugation and determined whether the binding of MEC-17 to MTs is affected by its phosphorylation status. Non-phosphorylated and phosphorylated MEC-17 cofractionated with the MT pellet in equal amounts (Figure 6c). Thus PAK1 does not regulate the binding of MEC-17 to MTs but probably acts on its catalytic site.

## 3. Discussion

Megakaryocytes are essential for hemostasis as these highly specialized cells produce the blood platelets. After maturation into a giant polyploid cell, they extend long PPLs from which platelets are released. The use of knockout mice models has highlighted essential functions for the small GTPases Rac and Cdc42 in megakaryocyte biogenesis and PPL formation [14]. PAK kinases are downstream effectors of Rac/Cdc42 and they regulate both MT and actin cytoskeletons through a number of targets. Like in the double Rac/Cdc42 knockout mice, PAK2^-/-^ MKs have defects in endomitosis, abnormal morphology and fail to form PPLs, thus resulting in macrocytopenia [14,16]. Here, using a cellular model that allows functional studies of PPL formation and elongation, we highlighted an essential role of another group I PAK, PAK1.

We show that in the absence of PAK1 activity, cells form several short protrusions instead of one or two long elongated PPLL as observed in control cells. PPL formation is mediated by MTs. They form cortical MT bundles that orient parallel to the plasma membrane and then dynein-mediated sliding of these MT bundles one against each other generates initial broad pseudopodia which further narrow and elongate to become PPLs [3,4]. MT bundles are predominantly acetylated but whether MT acetylation contributes to the bundling is still unclear [28]. We found that PAK1-depleted cells have increased MT acetylation levels. One possibility to explain the increased number of protrusions in PAK1-depleted cells is that MT acetylation favors the formation of bundled MTs and thus induces the formation of numerous pseudopodia. However, we show that only one or two PPLLs are formed in cells with hyperacetylated MTs generated by inhibition of HDAC6 activity. Thus, MT hyperacetylation is not sufficient to induce the formation of multiple protrusions seen in PAK1-depleted cells, and additional PAK1 substrates are likely required. Inhibition of HDAC6 activity also reveals that MT hyperacetylation does not prevent PPLL elongation. This observation is not unexpected since PPLL elongation depends on dynein-mediated MT sliding and several studies described MT acetylation as a positive regulator of dynein-based transport [29,30,31]. As MT hyperacetylation is not the cause of lack of PPLL elongation in PAK1-depleted cells, we propose that PPLL elongation could be limited by the amount of available demarcation membranes in D723H cells.

Both absence of PAK1 activity and inhibition of HDAC6 activity in D723H cells induce abnormally high MT acetylation levels and lead to the loss of MT integrity. Loss of integrity of hyperacetylated MTs has been reported earlier in neuronal and non-neuronal systems and has been proposed to be caused by increased sensitivity to the MT severing enzyme katanin [32,33,34,35]. It is not clear whether MT acetylation directly regulates the binding and activity of katanin to MTs since it has not been reproduced in vitro. The only direct effect of MT acetylation that has been described so far is a loss of inter-protofilament interactions [36,37]. Katanin was proposed to sever MTs by binding to the C-terminal tubulin tails and pulling on them to dislodge the tubulin from the MT lattice. Reduced inter-protofilament interactions might facilitate the work of katanin.

Whatever the mechanism by which MT hyperacetylation causes loss of MT integrity, it is clear that the MT acetylation levels need to be tightly regulated during PPL formation to preserve MT functionality and integrity. In this context, we identify PAK1 as a direct regulator of the acetyltransferase MEC-17. MEC-17 is highly conserved and the structure of its N-terminal catalytic core domain in complex with its cofactor acetyl-CoA has been resolved [38]. We show that PAK1 phosphorylates several sites of the unstructured C-terminal regulatory domain of MEC-17, inhibiting its acetyltransferase activity without affecting its binding to MTs. One possible mechanism is that PAK1 favors a “closed” conformation of MEC-17 in which the phosphorylated C-terminal tail interacts with the active site hindering the binding or hydrolysis of ATP required to catalyze the transfer of an acetyl-group to MTs. PAK1 had already been shown to act on MT dynamics by targeting the MT regulators stathmin or MCAK [39,40]. We now uncovered a novel pathway by which PAK1 regulates MT dynamics by directly phosphorylating MEC-17 and influencing the level of MT acetylation.

In conclusion, our study identifies PAK1 as an essential kinase during PPL formation. It maintains the correct MT acetylation level by directly phosphorylating the MT acetyltransferase MEC-17 thereby preserving MT integrity. Over the last couple of years, many PAK1 inhibitors have been developed and tested *in cellulo* for their anti-cancer potential. Indeed, PAK1 has gained interest as a promising target for cancer therapy as it was found to be frequently overexpressed in different types of cancer tissues and its abundance correlates with poor prognosis (for review see [41,42]). In the light of this study, the potential in vivo efficacy of PAK1 inhibitors on cancer cells might need to be counterbalanced by possible side effects on platelet formation.

## 4. Materials and Methods

### 4.1. Antibodies and Reagents

Rabbit antibodies against tubulin C102 was a gift of Dr. M. Andreu (Arevalo et al., 1990) and the antibodies against pE-Tubulin were home-made in rabbit (van Dijk et al., 2007). All other antibodies are commercially available: PAK1T212 (Abcam, Paris, France), α-tubulin YOL1/34 (Santa Cruz Biotechnology, Heidelberg, Germany), α-tubulin DM1A (Sigma, St Quentin Fallavier, France), Acetyl-tubulin 6-11B-1 (Santa Cruz Biotechnology, Heidelberg, Germany), Vinculin (Sigma, St Quentin Fallavier, France), Myc clone 9E10 (Developmental Studies Hybridoma Bank, Iowa City, USA), MEC-17 C6ORF134 (Sigma, St Quentin Fallavier, France), HDAC6 (Santa Cruz Biotechnology, Heidelberg, Germany), PhosphoT423-PAK1 PPAK1 (Cell Signaling, Leiden, The Netherlands). FRAX1036 was a kind gift of Dr. J. Chernoff (Fox Chase Cancer Center, Philadelphia, PA, USA). The other reagents were obtained from the indicated companies: hFibrinogen (Enzyme Research, Swansea, UK)**,** recombinant mouse thrombopoietin (Invitrogen, Thermo Electron SAS, Courtaboeuf, France), IPA-3 (Sigma, St Quentin Fallavier, France), Tubastatin A hydrochloride TBSA (MedChemExpress, Groningen, The Netherlands), Taxol (Sigma, St Quentin Fallavier, France), Trichostatin A TSA (Sigma, St Quentin Fallavier, France).

### 4.2. Megakaryocytes

Livers from embryos of a E13.5 pregnant OF1 were a gift of Dr. Boizet-Bonhoure (IGH, Montpellier, France) and were placed in RPMI medium. Megakaryocytes were differentiated for 4 days from the fetal liver cells with 50 ng/mL TPO as described [43]. Wild type pregnant OF1 females were obtained from Janvier Laboratories (Janvier Labs, Le Genest-Saint-Isle, France). Animal dissections were conducted according to procedures approved by the local and Regional Ethics committees (agreement number 34-366 to Dr. Boizet-Bonhoure).

### 4.3. Cell Lines, Cell Transfection and Drug Treatment

CHO cells expressing integrin α2bβ3-D723H (D723H cells) were a kind gift of Dr. N. Kieffer and were grown according to standard protocols. siRNAs (30 nM) were transfected for 48 h using RNAiMAX (Invitrogen) and plasmids for 24 h with Lipofectamine or Lipofectamine/plus reagent (Invitrogen). To induce PPLL formation, cells were serum starved (2 h), washed and seeded on fibrinogen-coated (50 µg/mL) glass-coverslips. Drugs were added 30 min to 2 h after seeding.

### 4.4. siRNA, Plasmids, Site Directed Mutagenesis

To deplete Luciferase (control), PAK1 and MEC-17 from D723H cells, we respectively used Eurogentec synthesized siRNA 5′-CGUACGCGGAAUACUUCGA(dT)(dT)3′, siRNA 5′-GCCCGAUUGCUUCAAACA(dT)(dT)3′ targeting nt310-328 from ATG of PAK1 and siRNA 5′-CCACACCAACUGGCCAUUGA (dT)(dT)3′, targeting nt478-497 from ATG of MEC-17. For mammalian cell transfection we used pCMV6M-PAK1 and pCMV6M-PAK1-K299R (Myc-PAK1-KD) that were gifts from Dr. J. Chernoff and are now commercially available (Addgene plasmids #12209 and # 12210). For recombinant PAK1 production in E. Coli, we used pMBP-X-PAK1-Cter (described in [44]). For MEC-17 production we started from a full length mouse MEC-17 cDNA in sPort–CMV vector (gift from Dr. J. Gaertig, University of Georgia, Athens, USA). Full-length MEC-17, its N-ter (aa 2-186) or its C-ter (aa 192-421) domains were amplified by PCR and subcloned into pGEX-4T-1 vector to add an N-terminal GST tag (Amersham). Site directed mutagenesis was performed using the QuickChange Site-directed mutagenesis kit (Agilent Technologies) on pGEX-4T-1-mMEC-17FL, all mutations were verified by sequencing. Oligonucleotide sequences used for PCRs are given in Table 1.

### 4.5. Immunofluorescence

Megakaryocytes or D723H cells were fixed with 4 % paraformaldehyde in PEM buffer (0.1 mM PIPES, pH 6.9; 1 mM EGTA; 0.5 mM MgCl2) containing 0.2 % Triton X-100 for 10 min at 37 °C or in MeOH for 10 min at -20 °C, blocked with 1 % BSA and stained with appropriate antibodies diluted in PBS containing 1% BSA for 1 h, then washed 5 times in PBS and incubated with Alexa 350, 488 and 555 conjugated secondary antibodies (Life Science) and when indicated with Alexa 647 conjugated phalloidin for actin visualization (Life Science). Cells were mounted in Mowiol with anti-fading N-Propyl Gallate.

### 4.6. Western Blot Quantification

Protein bands from western blots were quantified by densitometry using Image J. The amounts were normalized using either tubulin or vinculin as indicated on the figures.

### 4.7. Cell Imaging

Fixed cells were observed with wide field or confocal microscope as indicated. Wide field fluorescence microscopy was performed using a Zeiss AxioimagerZ1 microscope equipped with a scMOS ZYLA 4.2 MP camera and a 40X EC plan Neofluar 1.3NA oil objective. Confocal microscopy was performed using a Leica SP8-UV confocal microscope equipped with a Leica H63× HCX PL APO 1.4 oil CS2 objective. Most images are Maximal Intensity Projections (MIP) of 3D Z stacks. Sometimes single planes are shown, as indicated. For time lapse microscopy, images were recorded on an Olympus IX83 microscope equipped with Andor scMOS ZYLA 4.2 MP camera and a 20× AIR RC2 objective (0.45 numeral aperture) and driven by Metamorph software.

### 4.8. Image Quantification and Statistical Analyses

For quantification of colocalization, images were first thresholded using an automated threshold algorithm described in [45]. Then, Pearson correlation coefficients were calculated using Imaris Software (Bitplane, Oxford Instruments). Statistical significance of the measured Pearson coefficient was assessed using a simplified randomization algorithm based on the approach described in [45]. Briefly, channels intensities from one channel are randomly shuffled across the volume and the Pearson coefficient of the shuffled channel vs the second original channel is calculated. A distribution of “random” Pearson coefficient is then obtained and compared to the measured coefficient using both original channels. All obtained p values show that the measured Pearson coefficients could not be obtained with similar intensities “random” images. For quantification of the mean fluorescence intensities of acetyl-tubulin and total tubulin stainings as well as of cell shape descriptors (Perimeter and Aspect Ratio AR, ratio of major to minor cell axis lengths) we used Image J software. Images were first segmented using an intensity threshold and unresolved clusters of cells were discarded. Fluorescence intensity ratios of acetylated-tubulin to total-tubulin and perimeter and Aspect Ratio values were entered in Graphpad Prism software. The number of cells counted is indicated in figure legends. Statistical calculations were performed using a two-tailed unpaired Student’s *t* test. *p <* 0.0005 is considered significant. Error bars are SEM.

### 4.9. HDAC6 Activity Assays

Cells were lysed in PLB buffer (25 mM TRIS-HCl, pH 7.4, 150 mM NaCl, 5 mM EDTA, 1% Triton X-100, 10% glycerol, 1 mM NaF, 10 mM Sodium β-glycerophosphate, 1 mM DTT and protease inhibitor mix). After centrifugation, the cell lysates were mixed with magnetic protein A beads coupled to anti-HDAC6 antibodies. After a two-hour incubation at 4 °C, beads were washed three times in PLB buffer and then twice in PEM buffer. HDAC6-beads were further incubated for 1 h with 2 µg porcine brain MTs to test for HDAC6 activity, as previously described [46]. Pig brain tubulin was purified by two cycles of polymerization/depolymerization in high-molarity buffer as described [47] and polymerized into MTs in the presence of 25 µM taxol.

### 4.10. Recombinant Protein Purification

MBP-X-PAK1-Cter fusion proteins and GST-Mec17 fusion proteins were produced in E. Coli and purified according to the manufacturer’s recommendations (Biolabs and Thermofisher).

### 4.11. Xenopus CSF Extracts

Cytostatic factor (CSF) activity-arrested *Xenopus* egg extracts were prepared as described previously [48]. In brief, fresh eggs were dejellied in 2% cysteine (pH 7.8) and washed in XB buffer in the presence of 6 mM EGTA. Eggs were crushed at 20,000 *g* for 15 min, and the cytoplasmic layer was supplemented with an ATP regenerating system, cytochalasin B, and protease inhibitors. For MT sedimentation assays, the resulting CSF extract was further spun at 186,000 *g* for 2.5 h to generate high speed supernatants (HSS-CSF). Animal experiments were performed in the local Xenopus Facility (agreement number A3417239) and the procedures were approved by the local and Regional Ethics committees (APAFIS#19179-2019021415026143 v3).

### 4.12. In Vitro Kinase Reaction

Reactions were carried out as in [49] using active MBP-X-PAK1-Cter (0.5 µg) and GST-Mec17 full length or truncates (0.8µg) with γ-[33P] ATP in Kinase-buffer (25 mM HEPES, pH 7.5, 25 mM MgCl_2_, 25 mM Sodium β-glycerophosphate, 2 mM DTT, and 0.1 mM orthovanadate). Reactions were loaded on 10% SDS-PAGE, transferred to PVDF membranes, and visualized by autoradiography.

### 4.13. MEC-17 Activity in CSF Extract

In vitro phosphorylation was performed with Kinase-buffer/GST-MEC-17 (0.8 µg) and increasing MBP-X-PAK1-Cter protein (0-3 µg range) in the presence of gammaS-ATP (0.3 mM) to avoid phosphatase-mediated dephosphorylation. 30 % of the reactions were incubated in 30 µL CSF extract (30 min) and aliquots tested for MEC-17 activity with acetyl-tubulin as a readout.

### 4.14. MEC-17 Binding to MTs

In total, 0.8 µg of in vitro phosphorylated GST-MEC17 (by 1 µg of MBP-X-PAK1-Cter) was incubated in HSS CSF for 30 min. MTs were polymerized from HSS during 30 min at 25 °C, by adding 25 µM taxol. The extract was then centrifuged at 50,000× *g* for 20 min through a 40% Glycerol cushion in BRB80 Buffer and the MT-pellet (P) and soluble supernatant (S) fractions were analyzed for MEC-17 binding to MTs by western blot.

## Figures and Tables

**Table 1:** Sequences of Primers used for cloning and mutagenesis of MEC-17.

MEC-17 Cloning and Mutagenesis Primers	5′ → 3′ Sequence
Forward primer for cloning Mec-17 Full Length and N-ter	ccgcggatccgagttcccgttcg
Reverse primer for cloning Mec-17 Full Length and C-ter	tccgctcgagttaccaaggcctggtgctgc
Forward primer for cloning Mec-17 C-ter	ccgcggatcccatcagcaccggccccc
Reverse primer for cloning Mec-17 N-ter	tccgctcgagttaaaagatgacaaagttgttcacc
Forward primer T260A	ggcccctcggcgtgccGCAcctccagc
Reverse primer T260A	ggtgggtgggctggaggTGCggcacgc
Forward primer S270A	cccacccacctccacgtGCTagcagcctgg
Reverse primer S270A	gagttgcccaggctgctAGCacgtggagg
Forward primer S271A	cccacctccacgttctGCCagcctgggc
Reverse primer S271A	ggtgagttgcccaggctGGCagaacgtgg
Forward primer S272A	ccacctccacgttctagcGGCctgggc
Reverse primer S272A	ccggtgagttgcccagGCCgctagaacg
Forward primer S276A	ctagcagcctgggcaacGCAccggatcg
Reverse primer S276A	gggaccccgatccggTGCgttgcccagg
Forward primer S315A	ccactgaccctggaggcGCCccagccc
Reverse primer S315A	cgtctgcgctgggctggGGCgcctccagg

## Supplementary Materials

Supplementary materials can be found at https://www.mdpi.com/1422-0067/21/20/7531/s1.

## Abbreviations

Ac-tubulin	Acetylated tubulin
BSA	Bovine serum albumin
CHO	Chinese hamster ovary
DMS	Demarcation membrane system
HDAC6	Histone deacetylase 6
LUC	Luciferase
MIP	Maximum intensity projection
MK	Megakaryocyte
MT	Microtubule
PAK1	P21-activated kinase
pE-tubulin	Polyglutamylated tubulin
PPL	Proplatelet
PPLL	Proplatelet-like
SEM	Standard error of the mean
TBSA	Tubastatin A hydrochloride
TPO	Thrombopoietin
TSA	Trichostatin A
