# Supplementary figures and images for: PAK1 Regulates MEC-17 Acetyltransferase Activity and Microtubule Acetylation during Proplatelet Extension
Source: Int J Mol Sci. 2020 Oct 13;21(20):7531. doi: 10.3390/ijms21207531 (PMC7589885; doi:10.3390/ijms21207531)

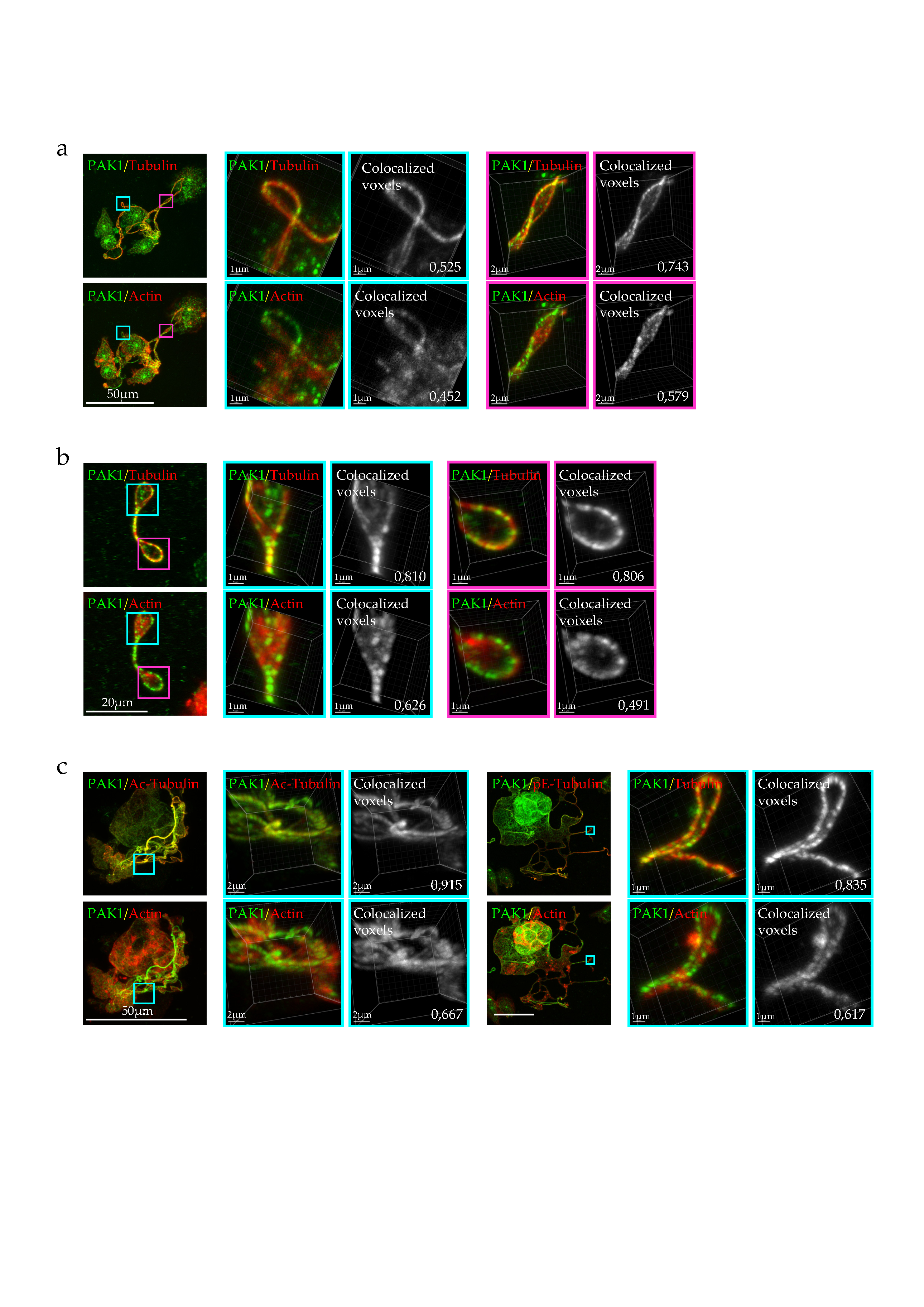

Supplement: Supplementary file 1 [file ijms-21-07531-s001.zip › Suppl. Fig 1.tiff]

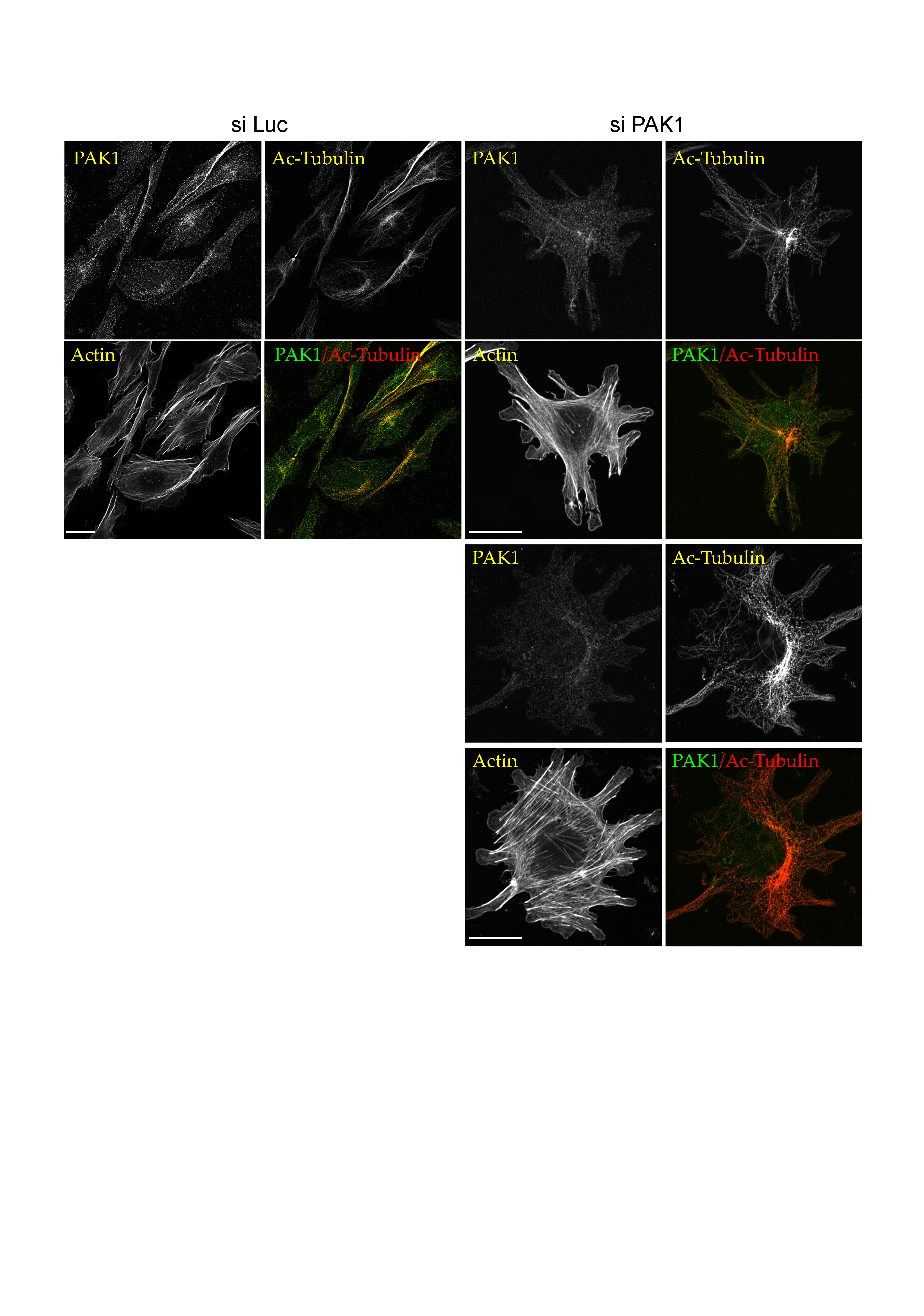

Supplement: Supplementary file 1 [file ijms-21-07531-s001.zip › Suppl. Fig 2.tiff]
